# Flow-through Omental Flap for Vascularized Lymph Node Transfer: A Novel Surgical Approach for Delayed Lymphatic Reconstruction

**DOI:** 10.1097/GOX.0000000000002436

**Published:** 2019-09-30

**Authors:** Anna Rose Johnson, Miguel G. Bravo, Melisa D. Granoff, Christine O. Kang, Jonathan F. Critchlow, Leo L. Tsai, Bernard T. Lee, Dhruv Singhal

**Affiliations:** From the * Division of Plastic and Reconstructive Surgery, Department of Surgery, Beth Israel Deaconess Medical Center, Harvard Medical School, Boston, Mass.; † Department of Surgery, Beth Israel Deaconess Medical Center, Harvard Medical School, Boston, Mass.; ‡ Department of Radiology, Beth Israel Deaconess Medical Center, Harvard Medical School, Boston, Mass.

## Abstract

The vascularized omental free flap has been described as a reliable option for the treatment of peripheral lymphedema. However, the flap has been associated with venous hypertension which may require venous supercharging or intra-flap arteriovenous fistula creation to offload the arterial inflow. The aim of this study is to introduce and present our experience using a flow-through omental flap as a novel approach to optimize flap hemodynamics. A retrospective review of a prospectively maintained quality improvement database was performed. Seven consecutive patients with unilateral breast cancer-related lymphedema (BCRL) who underwent delayed lymphatic reconstruction using a flow-through omental free flap were identified. In all patients, the right gastroepiploic artery and vein were anastomosed to the proximal end of the radial artery and to one venae comitante, respectively. An anastomosis of the distal end of the radial artery to the left gastroepiploic artery was performed. The flap was then supercharged by anastomosing the left gastroepiploic vein to the cephalic or basilic vein. There were no flap losses or other surgical complications. A distinct advantage of this inset includes the ability to moderate the arterial in-flow to the omental flap to avoid an inflow-outflow mismatch and alleviate venous hypertension. Further study is needed to validate this technique in a larger study sample with longer follow-up.

## Abstract 2

Supplemental Digital Content is available in the text.

## INTRODUCTION

Vascularized lymph node transfer (VLNT) has emerged as a promising surgical approach for the treatment of chronic lymphedema (LE), associated with both quantitative and subjective improvements.^1^ However, there remain concerns regarding its potential to cause donor-site LE, which has been described in 1.3%–23.1% of patients.^2,3^ The use of the omentum for VLNT has increased in popularity due to its angiogenic and immunologic properties.^4^ Further, it has no risk of donor-site LE.^5^ However, a remaining concern with the omental flap is its association with intra-flap venous hypertension.

A single arterial and venous anastomosis for the omental flap has been reported to increase the risk of venous hypertension.^6^ To relieve venous congestion, techniques such as venous supercharging or the creation of an intra-flap arteriovenous fistula have been proposed.^4,6^ However, technical modifications to facilitate an optimal pressure gradient to maintain adequate lymph perfusion have not been determined.

The addition of a distal arterial anastomosis has the potential to offload potential venous congestion by creating a better arteriovenous flap gradient. In this paper, we aim to propose a novel surgical technique to decrease venous hypertension and describe our initial experience using the omentum as a flow-through flap for the treatment of patients with chronic breast cancer-related lymphedema (BCRL).

## METHODS

A retrospective review of a prospectively maintained quality improvement database was performed. Institutional Review Board approval was obtained. Consecutive patients with unilateral BCRL who underwent a flow-through omental free flap were identified. Patient characteristics, intraoperative specifics, and postoperative outcomes, including quality of life metrics, were retrieved.

### Preoperative Considerations

The selection of the recipient location was determined by physical exam and preoperative imaging. Allen testing was performed preoperatively and a positive test or a history of damage to the vascular arch would be a relative contraindication to the arterial inset described below. In this scenario, the surgeon can consider an alternate site for flap inset or an end-to-side configuration.

### Surgical Technique

The omental flap was harvested in collaboration with general surgery (J.F.C). The abdomen was accessed using an 8-cm supraumbilical incision and dissection to the omentum was performed. The lesser sac was then entered and the omentum was exposed and dissected away from the transverse mesocolon. A radiologist (L.L.T.) used intraoperative ultrasound (IOUS) to identify and quantify lymph nodes as previously described.^7^ The omentum was harvested and the proximal and distal gastroepiploic vessels were identified.

**Fig. 1.**
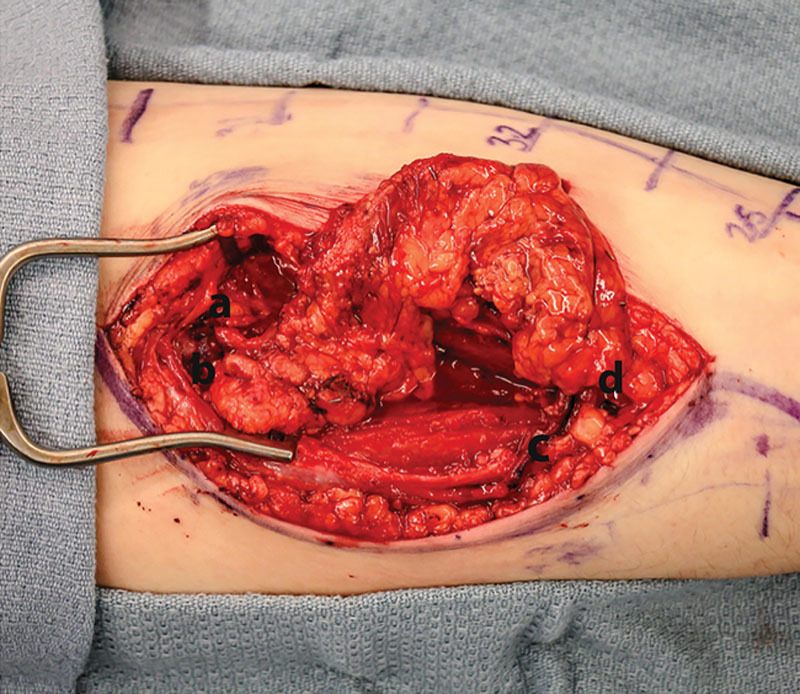
Intraoperative image illustrating flow-through omental flow insetting.

The forearm was simultaneously prepared for flap inset using a 10-cm curvilinear incision fashioned over the course of the radial artery and venae comitantes. Dissection proceeded through the superficial soft tissue with care, preserving the superficial sensory nerves. The brachioradialis was reflected to expose the radial vessels. The right gastroepiploic artery and vein were anastomosed to the proximal end of the radial artery and to 1 venae comitante, respectively. The distal end of the radial artery was anastomosed to the left gastroepiploic artery. The flap was then supercharged by anastomosing the left gastroepiploic vein to the cephalic vein (Fig. 1). To improve potential lymphangiogenesis, deep fat and muscle fascia from the radial and ulnar aspects of the forearm were removed.^4^ All patients were closed primarily in a layered fashion after flap inset and confirmation of patency. A transcutaneous doppler was used for postoperative flap monitoring (see Video 1 [online], for a schematic illustration of surgical technique).

**Video 1.** This Video displays illustration of the flow-through omental flap surgical technique.

## RESULTS

Seven consecutive patients with unilateral BCRL underwent a flow-through free omental flap to the forearm by a single surgeon (D.S.) from January 2018 to April 2019 (Table 1). All patients were females with a mean age of 56.9 years (41–73) and body mass index of 27.8 kg/m^2^(24.4–32.3). All were classified with stage II LE by certified LE therapists using the International Lymphedema Staging criteria. Mean flap weight was 25.7 g (16–40) and mean number of lymph nodes transferred detected by IOUS was 7.3 (6–11). The mean length of the omental flap was 12 cm (9–14) and mean width of 3 cm (3–4). Mean tissue weight debulked was 22 g (12–34). The mean difference between the flap weight and the debulked tissue was 3.6 g and ranged from −12 to 14 g. Mean coupler size for the left gastroepiploic vein was 2.3 mm (2–2.5), while the right gastroepiploic vein was 3 mm (2–3) (Table 2). Postoperative outcomes for patients with a minimum of 3 months of follow-up demonstrated significant improvement in lymphedema quality of life (LYMQOL) domains with mean percent increases of 25% (mood), 23% (symptoms), 33% (function), and 30% (appearance). The quality of life domain score increased by a mean of 27%.

**Table 1.** Patient Characteristics and Surgical Specifics

Patient ID	Age (y)	BMI (kg/m^2^)	Diagnosis	Prior surgical treatment	Flap dimensions (cm)	Flap weight (g)	Lymph nodes detected by IOUS	Additional; debulked tissue weight (g)	Coupler size—left gastroepiploic vein (mm)	Coupler size—right gastroepiploic vein (mm)	Length of stay (d)
1	73	25.1	R. BCRL	Lumpectomy/ALND/XRT*/chemotherapy	14 × 3	22	6	34	2.0	2.0	6
2	68	29.9	L. BCRL	Mastectomy/ALND/XRT/chemotherapy	15 × 3	40	11	26	2.0	2.5	5
3	51	32.3	R. BCRL	Lumpectomy/ALND/XRT/chemotherapy	14 × 3	24	7	28	2.5	2.0	4
4	65	31.0	R. BCRL	Mastectomy/ALND/XRT/chemotherapy	9 × 4	32	6	22	2.5	2.5	3
5	41	25.8	L. BCRL	Mastectomy/ALND/XRT/chemotherapy	11 × 3	32	8	20	2.5	2.0	4
6	47	24.4	R. BCRL	Lumpectomy/ALND/XRT/chemotherapy	12 × 3	16	6	12	2.0	3.0	4
7	53	26.3	L. BCRL	Mastectomy/ALND/XRT/chemotherapy	10 × 3	14	7	13	2.5	2.0	3

* Adjuvant radiation.

BMI, body mass index; ALND, axillary lymph node dissection; XRT, radiation treatment.

**Table 2.** Patient Quantitative Limb Measurement Changes

Patient ID	Excess volume (preoperative)	Excess volume (postoperative)	Change in excess volume (%)	L-Dex (preoperative)	L-Dex (postoperative)	Change in L-Dex (units)	Follow-up (mo)
1	262	65	−76.3	12.8	6.7	−6.1	5
2	574	366	−36.24	30.9	14.9	−16	12
3	1,149	775	−32.55	55.9	36.5	−19.4	11
4	300	287	−4.53	7.8	5.9	−1.9	14
5	368	157	−57.3	27.5	18.2	−9.3	9
6	201	230	+14.3	8.1	NA	NA	3
7	562	774	+33.7	24.7	25.8	+1.1	1
Mean	488	379.1	−22.7	25.9	18	−8.6	7.8

Mean follow-up time was 7.9 months (1–14) (Table 2). The mean excess volume reduction was 22.7%. When examining patients with a minimum of 6-month follow-up, the mean excess volume reduction was 41.3%. Mean decrease in L-dex value was 8.6 units (n = 6 patients) (Table 2). Pre- and postoperative images for a patient undergoing VLNT are provided (see figure, Supplemental Digital Content 1, which displays preoperative and postoperative images for a patient undergoing flow-through VLNT, http://links.lww.com/PRSGO/B222).

**Fig. 2.**
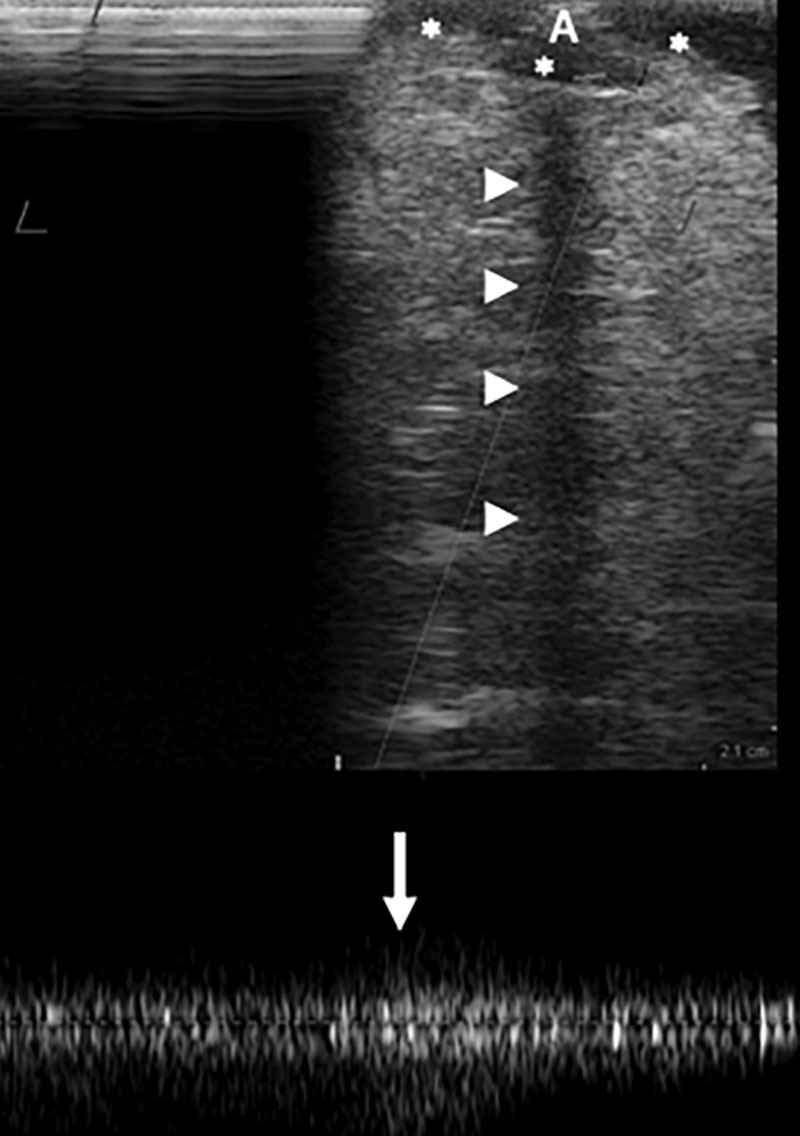
Intraoperative US evaluation of the supercharged basilic vein following transplantation. The imaging depth is 2.1 cm. The vein (*) lies at the surface. Posterior shadowing (arrowheads) is caused by the coupler anastomosis (A). Venous patency is demonstrated using spectral Doppler (bottom). Low pressure flow in the basilic vein is demonstrated with augmentation (arrow) via gentle compression from the flap.

**Fig. 3.**
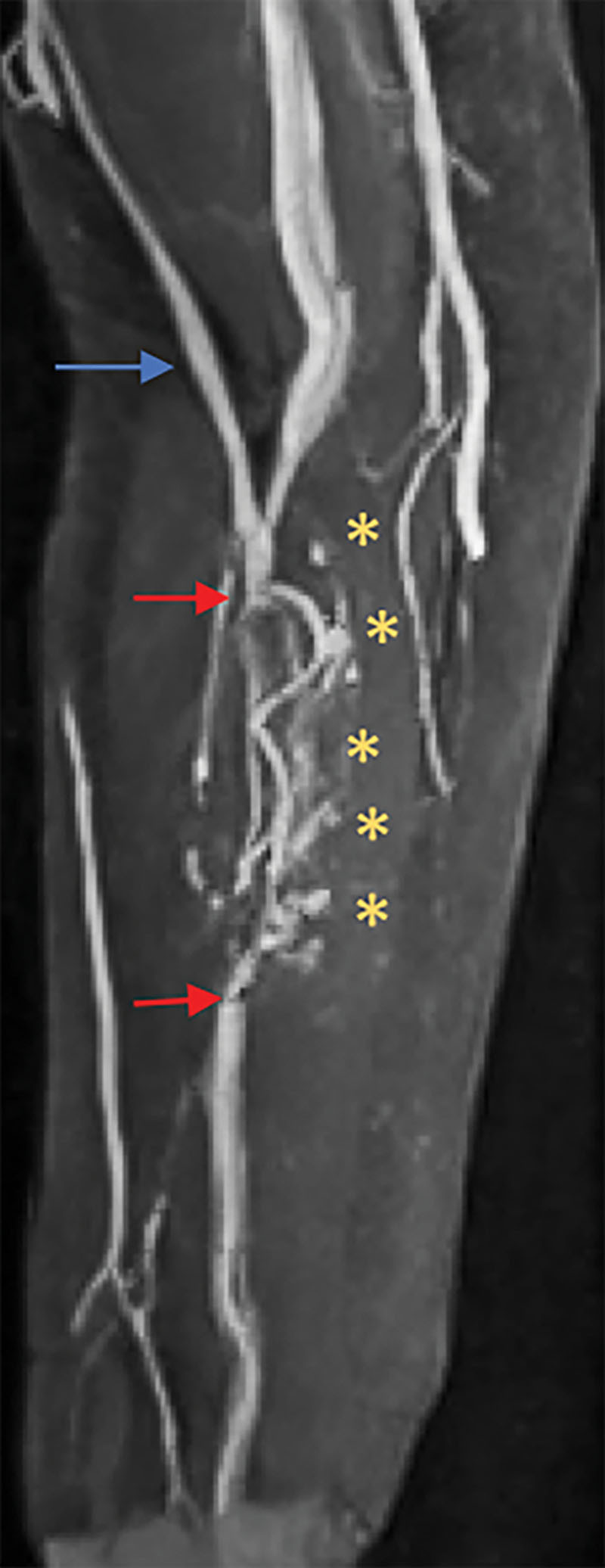
Postoperative MRA imaging of an operative extremity after flap inset. The blue arrow reflects the location of the cephalic vein. The red arrows correspond to the arterial anastomoses. The asterisk (*) denotes lymph nodes.

The intraoperative technique illustrating gradual arterial perfusion and a low-flow venous pressure system is provided [see Video 2 [online], which displays intraoperative video illustrating flow-through omental flap; yellow arrow indicates the region of the proximal anastomoses (proximal radial artery and associated venae comitantes to the gastroepiploic system) and the blue arrow indicates the supercharged venous anastomotic site (basilic vein to the left gastroepiploic vein). The red asterisks indicate lymph nodes identified through IOUS. The dashed blue line indicates the low flow through that can be seen in the vein.] Doppler signal confirmed excellent flow in all patients. There were no perioperative or postoperative complications reported including venous congestion or flap failure. An IOUS demonstrating intraoperative venous patency using spectral Doppler can be seen in Figure 2. All flow-through omental flaps were soft on clinical exam in both initial and latest follow-up. An example of a postoperative magnetic resonance angiography (MRA) illustrating the flap vasculature and lymph nodes is demonstrated in Figure 3. See Supplemental Digital Content 2 for our institutional postoperative therapy regimen. (See figure, Supplemental Digital Content 2, which displays postoperative therapy regimen for patients undergoing VLNT, http://links.lww.com/PRSGO/B223.)

**Video 2.** This Video displays intraoperative video illustrating flow-through omental flap; Yellow arrow indicates the region of the proximal anastomoses (proximal radial artery and associated venae comitantes to the gastroepiploic system) and the blue arrow indicates the supercharged venous anastomotic site (basilic vein to the left gastroepiploic vein). The red asterisks indicate lymph nodes identified through IOUS. The dashed blue line indicates the low flow through that can be seen in the vein.

## DISCUSSION

In this study, we present a novel surgical approach using a flow-through omental flap for the treatment of BCRL and review our surgical experience. Specifically, we believe this approach optimizes arterial dynamics by decreasing flap inflow to minimize venous hypertension while preserving physiologic perfusion to the distal extremity.

While the capillary networks of traditional free tissue transfers (eg, rectus abdominus muscle) are quite extensive, allowing for equilibration of inflow and outflow mismatches, lymph node free tissue transfers are primarily composed of lymph nodes and fat with less defined capillary networks. Thus, given the cascade anatomy, an optimal relationship between inflow and outflow is paramount. In prior studies, an end-to-side anastomosis of free tissue in patients with peripheral vascular disease resulted in a steal phenomenon.^9^ That is, arterial inflow to the transferred tissue was significant enough to result in decreased peripheral flow to the extremity. A porcine study of the omental flow-through flap demonstrated that flow dynamics were unchanged at the distal arterial anastomosis^7^ even when the omental flap was clamped.^10^ Thus, in a flow-through configuration, the arterial inflow to the flap is significantly less than in an end-to-side configuration.

In the setting of a lymph node transplantation, we believe the omental flap flow-through configuration has the capacity to autoregulate arterial inflow to the lymph nodes minimizing the risk of venous hypertension. This theory is consistent with previous studies which have provided evidence for the hemodynamic benefits of the flow-through flap to offset venous congestion.^10,11^

The flow-through omental free flap to the forearm may be considered as a reliable surgical option for patients with BCRL. Further study is needed to validate this novel technique and clinical efficacy in a patient population with longer follow-up.

## Supplementary Material
