# Supplementary material for: Flow-through Omental Flap for Vascularized Lymph Node Transfer: A Novel Surgical Approach for Delayed Lymphatic Reconstruction
Source: Plast Reconstr Surg Glob Open. 2019 Sep 30;7(9):e2436. doi: 10.1097/GOX.0000000000002436 (PMC6799400; doi:10.1097/GOX.0000000000002436)
Supplement: Supplementary file 3 [file gox-7-e2436-s003.pdf]

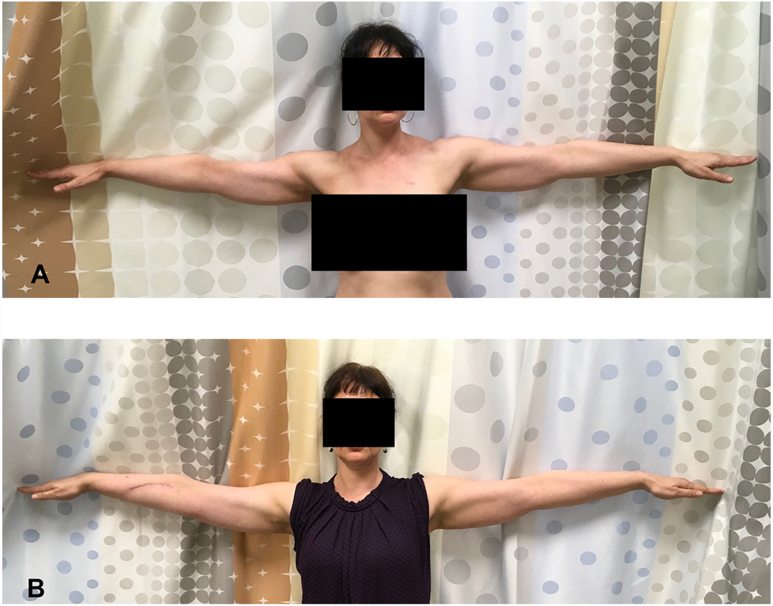

A preoperative [A] and post-operative image [B] of a patient who underwent a flow-through VLNT with fewer than 6 months follow up.
