# Supplementary material for: Flow-through Omental Flap for Vascularized Lymph Node Transfer: A Novel Surgical Approach for Delayed Lymphatic Reconstruction
Source: Plast Reconstr Surg Glob Open. 2019 Sep 30;7(9):e2436. doi: 10.1097/GOX.0000000000002436 (PMC6799400; doi:10.1097/GOX.0000000000002436)
Supplement: Supplementary file 4 [file gox-7-e2436-s004.pdf]

Immediate Postoperative Care

- No compression therapy
- Elbow placed in posterior splint; limb elevation emphasized
- No heavy exercise

Week 3-Month 3

- Initiate compression banging using standard multi-layer bandaging technique (stockinette, gauze padding, and short stretch bandaging)
- Eventual transition to compression garment before month 3
- Standard measurements (volumetry, bioimpedance spectroscopy)

Month 3 onward

- Repeat measurements; assess volume stabilization
  - Measurements stabilize→ reduce number of hours in compression
  - Measurements still not stabilized→ continue compression

All delayed lymphatic reconstruction patients are monitored by certified lymphedema therapists and have preoperative and post-operative measurements taken at prescribed intervals.
